# Community Perspectives and Behavior Change Strategies for Integrated Long-Acting Reversible Contraception and Long-Acting Injectable Preexposure Prophylaxis Roll Out for Adolescent Girls and Young Women in Uganda (COMPASS): Protocol for a Mixed Methods Implementation Science Study

**DOI:** 10.2196/88635

**Published:** 2026-09-11

**Authors:** Phionah Kibalama Ssemambo, Rita Nakalega, Benjamin H Chi, Nelson Mukiza, Juliane Etima, Clemensia Nakabiito, Mary Glenn Fowler, Andrew Mujugira

**Affiliations:** 1 Makerere University Johns Hopkins University (MU-JHU) Research Collaboration Kampala Uganda; 2 School of Medicine, University of North Carolina Chapel Hill, NC United States; 3 Rinecynth Advisory Limited Kampala Uganda; 4 Department of Pathology Johns Hopkins School of Medicine, Johns Hopkins University Baltimore, MD United States; 5 The Infectious Diseases Institute, Makerere University Kampala Uganda; 6 Department of Global Health University of Washington Seattle, WA United States

**Keywords:** adolescent girls and young women, AGYW, long-acting reversible contraception, LARC, long-acting injectable preexposure prophylaxis, LAI-PrEP, HIV prevention, reproductive health, implementation science, Uganda

## Abstract

**Background:**

HIV incidence among adolescent girls and young women (AGYW) aged 15-24 years in Eastern and Southern Africa remains unacceptably high. Unprotected sex increases the risk of HIV and other sexually transmitted infections and unintended pregnancies, reflecting an unmet need for effective contraception among AGYW. Long-acting injectable preexposure prophylaxis (LAI-PrEP) and long-acting reversible contraception (LARC) offer effective, durable protection against HIV and unintended pregnancy. However, uptake and persistence remain suboptimal due to low awareness, fear of side effects, stigma, limited decision-making autonomy, and poor integrated reproductive health and HIV prevention services. Evidence on community-driven and behaviorally informed strategies to support integrated rollout and uptake of LAI-PrEP and LARC among AGYW in Uganda remains limited.

**Objective:**

The study aims to determine the preferences and willingness of AGYW in Uganda to use LARC and LAI-PrEP, and characterize stakeholder perceptions, practices, and social norms that may influence adoption of integrated LARC and LAI-PrEP services. It applies the Capability, Opportunity, Motivation–Behavior (COM-B) model to identify facilitators and barriers to uptake and adherence.

**Methods:**

This mixed methods implementation science study, guided by the COM-B model and the Behavior Change Wheel (BCW), will be conducted in 3 Kampala Capital City Authority clinics to generate evidence and co-design community-driven strategies supporting integrated LARC and LAI-PrEP uptake among AGYW. The study will proceed in three stages: (1) structured surveys with 220 AGYW to assess determinants of uptake and willingness to use integrated services, (2) focus group discussions with 30 AGYW and in-depth interviews with 20 key informants to explore perceptions, preferences, and barriers, and (3) participatory co-design of behavior change strategies. Survey data will be analyzed using descriptive and multivariable regression methods, while qualitative data will undergo reflexive thematic analysis guided by Braun and Clarke. Findings will be integrated using a convergent mixed methods approach through triangulation and joint displays to identify factors influencing implementation of integrated LARC and LAI-PrEP services.

**Results:**

The study was funded in February 2026. Ethical approval was obtained from the Joint Clinical Research Centre Research Ethics Committee (JCRC IRB/EC; JCRC-2025-143), Kampala Capital City Authority (DPHE/KCCA/1302/01), and the Uganda National Council for Science and Technology (UNCST; HS6232ES). Written informed consent was obtained from all participants. Study preparation, including staff training, development of study materials, and community sensitization, was completed before participant recruitment. Participant recruitment began in March 2026, and was completed in July 2026, with 220 AGYW and 20 key informants enrolled. Data cleaning and quality assurance are ongoing, with quantitative and qualitative analyses planned following completion of all study activities. Findings are anticipated to be reported in 2027.

**Conclusions:**

Findings will provide evidence-based recommendations to inform the targeted interventions and policy guidelines for scaling up integrated LARC and LAI-PrEP services.

**International Registered Report Identifier (IRRID):**

PRR1-10.2196/88635

## Introduction

### Background

Adolescent girls and young women (AGYW) in sub-Saharan Africa (SSA) face a dual burden of unintended pregnancies and HIV, which significantly impacts their health, educational attainment, and socioeconomic opportunities [1]. Uganda has one of the highest fertility rates globally (4.3 children/woman) [2,3], with a substantial proportion of pregnancies occurring among AGYW [4-6]. Concurrently, this demographic accounts for a disproportionate number of new HIV acquisitions driven by gender-based violence, early marriage, transactional sex, limited access to sexual and reproductive health (SRH) services, stigma, and socio-economic disparities [7-10]. In Uganda, HIV prevalence among AGYW aged 15-24 years is 9.1%, with an estimated 11,000 of the 37,000 new HIV acquisitions in 2024 [11] occurring in this age group [8]. Across SSA, HIV incidence among AGYW remains high, approximately 3.92 per 100 person-years, highlighting the urgent need for targeted prevention strategies, including preexposure prophylaxis (PrEP), comprehensive SRH services, and community-driven interventions addressing structural and behavioral risk factors [12]. Despite the availability of effective HIV prevention tools such as oral PrEP, uptake and adherence remain suboptimal among AGYW due to stigma, forgetfulness, limited awareness, and constrained service delivery models [13-16]. Structural barriers within the health system further compound these individual and social challenges.

Delivering HIV prevention services effectively in public health settings is often complicated by health system barriers, including limited provider training, stock-outs of essential commodities, and stigma associated with PrEP [14,17]. At the same time, unintended pregnancies remain a persistent challenge, reflecting gaps in contraceptive access, knowledge, cultural norms, financial constraints, and limited decision-making autonomy among AGYW [5,18-22]. Leveraging established health care platforms, such as family planning clinics and other youth-friendly service points, offers an opportunity to integrate HIV prevention and reproductive health services, making care more accessible and acceptable to AGYW.

Long-acting interventions, including long-acting reversible contraception (LARC) and long-acting injectable preexposure prophylaxis (LAI-PrEP), offer promising, durable protection against unintended pregnancy and HIV acquisition among AGYW [15,16,23-29]. Integrating these services within existing, youth-friendly, community-based platforms, such as family planning clinics, could overcome logistical and social barriers, improve uptake, adherence, and reproductive health outcomes, and help normalize preventive health behaviors [30-39]. Successful integration requires understanding AGYW preferences and engaging key stakeholders, including peers, parents, partners, community leaders, and health care providers, to ensure services are youth-friendly, acceptable, and responsive to community needs [40-43]. Community-centered, behaviorally informed approaches that leverage trusted influencers have been shown to improve access, acceptability, and adherence to health interventions, including HIV prevention [44,45], aligning with World Health Organization (WHO) and Joint United Nations Programme on HIV and AIDS (UNAIDS) recommendations for community-led strategies as essential for achieving universal health coverage and the 95-95-95 HIV targets by 2030 [7,46,47]. Understanding community perspectives, preferences, and norms is critical to designing effective behavior change strategies that support integrated service delivery. In this protocol paper, we describe an implementation study designed to evaluate community-driven strategies to optimize the uptake and adoption of integrated LARC and LAI-PrEP services among AGYW in Uganda.

### Conceptual Framework

**Figure 1:**
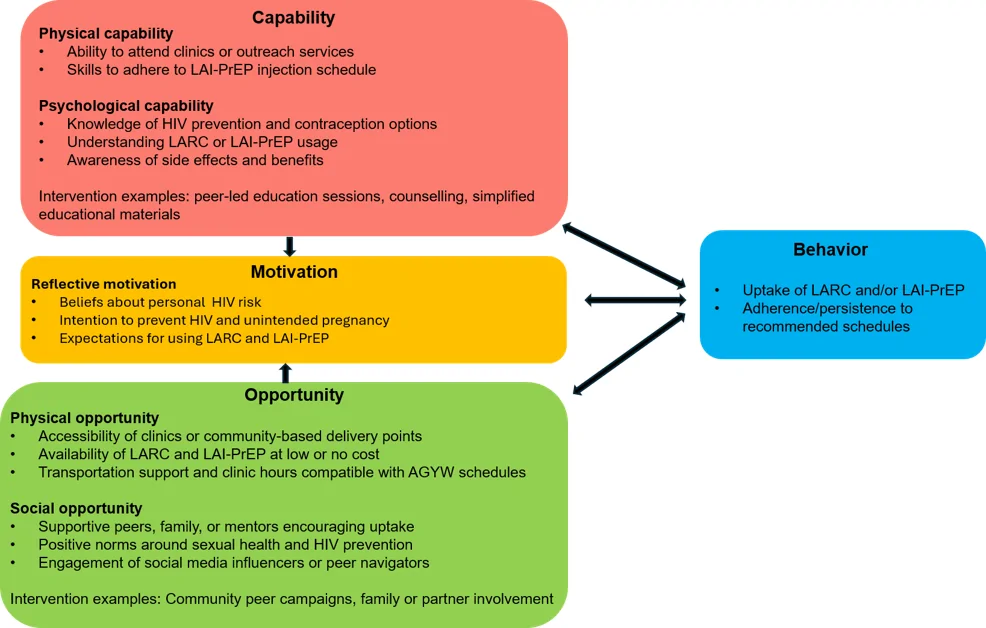
Adapted Capability, Opportunity, Motivation-Behavior (COM-B) model and the Behaviour Change Wheel (BCW) conceptual framework for the COMPASS study. The figure illustrates how Capability, Opportunity, and Motivation interact to influence uptake of integrated long-acting reversible contraception and long-acting injectable preexposure prophylaxis services among adolescent girls and young women in Uganda and how these behavioral determinants inform the development of behavior change strategies. This figure was generated using an artificial intelligence tool (OpenAI DALL·E). Prompt used: “Create a simple, non-photorealistic diagram illustrating the COM-B model (Capability, Opportunity, Motivation → Behavior) adapted for the COMPASS study.” Source link to the generated image [50]. AGYW: adolescent girls and young women; LARC: long-acting reversible contraception; LAI-PrEP: long-acting injectable preexposure prophylaxis.

Addressing the barriers to integrated LARC and LAI-PrEP uptake requires a systematic understanding of the behavioral and context-specific factors that influence service use. The Capability, Opportunity, Motivation-Behavior (COM-B) model and the Behavior Change Wheel (BCW) frameworks [48,49] provide this theoretical foundation by identifying determinants of behavior and linking them to appropriate intervention functions and implementation strategies. This model posits that behavior (in this case, uptake and adherence to integrated LARC and LAI-PrEP services) results from the dynamic interaction of Capability, Opportunity, and Motivation: (1) Capability encompasses AGYW’s knowledge, skills, and self-efficacy to use LARC and LAI-PrEP correctly and consistently, (2) Opportunity refers to external factors such as access to youth-friendly health services, provider support, peer influence, and community norms that facilitate or hinder uptake, (3) Motivation includes the internal processes that drive decision-making, such as perceived risk of HIV, attitudes toward contraception and PrEP, and willingness to use dual methods. These constructs are interlinked and inform each stage of study design, from participant recruitment and data collection tools to analysis and interpretation. The BCW will complement the COM-B model by identifying potential intervention functions (eg, education, persuasion, and enablement) and policy levers that can be used to address identified barriers and strengthen facilitators to uptake and adherence. The adapted conceptual framework for the COMPASS study is presented in Figure 1 [49], illustrating how COM-B components interact to influence behavioral outcomes related to the adoption of integrated LARC and LAI-PrEP services among AGYW in Uganda.

### Objectives

This study seeks to understand the uptake and adoption of integrated LARC and LAI-PrEP services among AGYW aged 15-24 years in Uganda, to inform community-driven strategies to optimize service delivery.

Specifically, the study aims to (1) determine AGYW preferences for and willingness to use LARC and LAI-PrEP, and (2) characterize stakeholder perceptions, practices, and norms, including those of AGYW peers, social media influencers, health care professionals, community leaders, and representatives from reproductive health and HIV prevention organizations that influence the adoption of integrated LARC and LAI-PrEP services.

## Methods

### Study Design

The COMPASS study will use a mixed methods, cross-sectional implementation science design. This study will use 3 data collection approaches (structured surveys, focus group discussions [FGDs], and in-depth interviews [IDIs]) to generate both quantitative and qualitative evidence on integrated LARC and LAI-PrEP services. Initially, quantitative data will be collected through structured surveys administered to AGYW attending selected health facilities in Kampala to assess their attitudes, preferences, and willingness to use LARC and LAI-PrEP (Multimedia Appendix 1). Qualitative data will be gathered through FGDs among a subset of surveyed AGYW to explore further perceptions and motivations influencing uptake in a group setting (Multimedia Appendix 2), and through IDIs with key stakeholders, including health care providers, community leaders, and program implementers, to provide detailed perspectives on the social, cultural, and health system factors that may influence the adoption of integrated services. Taken together, these approaches provide a layered understanding of both AGYW’s preferences and the broader contextual factors shaping uptake (Multimedia Appendix 3).

### Study Setting

The study will be conducted at 3 Kampala Capital City Authority (KCCA) clinics that currently provide both oral PrEP and LARC services: Kisenyi, Kawaala, and Komamboga Health Centers. Kisenyi Health Center, located in the heart of central Kampala, serves a densely populated urban area and primarily serves low-income AGYW who often face stigma, financial barriers, and limited access to reproductive health services. This urban setting enables the examination of health care preferences and barriers that are specific to densely populated city environments. Kawaala Health Center, located in a periurban area, serves communities that experience both urban and rural dynamics. AGYW in this catchment frequently encounter transportation challenges, reduced access to health care facilities, and socio-economic inequalities. Studying this periurban context provides critical insights into the barriers faced by AGYW in areas where health care needs are growing, but access remains limited. Komamboga Health Center, located on the northern outskirts of Kampala, serves a suburban population. AGYW in this area navigate challenges, including limited health care infrastructure and cultural barriers to adopting contraceptive and HIV prevention services. This suburban context provides a contrasting perspective to urban and peri-urban settings. These health centers were chosen due to their existing maternal and reproductive health programs, including youth-friendly services, making them ideal sites for piloting the integration of LARC and LAI-PrEP services in future interventions. Their diverse urban, suburban, and periurban contexts will enable the study to capture a representative range of AGYW experiences, challenges, and cultural contexts. Collectively, the findings from these centers are expected to generate actionable evidence to inform scalable models for integrating LARC and LAI-PrEP services across Uganda.

### Study Population

The study will enroll AGYW aged 15-24 years who meet the inclusion criteria outlined in Table 1. To align with the study objectives, eligibility is limited to AGYW who are current LARC users or have an existing interest in LARC, thereby focusing the study on a population for whom integrated LARC and LAI-PrEP services are most relevant while allowing assessment of variation in awareness, perceptions, and willingness to use LAI-PrEP. Up to 220 AGYW will be recruited across the three study sites to participate in the quantitative component through structured surveys. A subset of the AGYW participants will be invited to join up to three FGDs, each with approximately 10 AGYW, to explore further the attitudes, preferences, and willingness to use integrated LARC and LAI-PrEP services. In parallel, up to 20 key informants (KIs) who are stakeholders in reproductive health and HIV prevention and who meet eligibility criteria will participate in IDIs to characterize stakeholder perceptions, practices, and norms that influence the adoption of integrated services. The KI sample will include up to three individuals from each of the following groups, specifically: AGYW peers (including sex workers and social media influencers), local health authorities, health care professionals, community leaders, representatives from reproductive health and HIV prevention organizations, and representatives from civil society. These stakeholders were selected for their direct involvement in shaping service delivery.

Exclusion criteria include any condition that, in the opinion of the study team, would preclude informed consent, make study participation unsafe, complicate interpretation of study outcome data, or otherwise interfere with achieving the study objectives. This general exclusion applies to all participant groups. For AGYW survey participants only, additional exclusions include known HIV-positive status, confirmed through the most recent health record (eg, health passport, HIV test card, or similar document) or by self-report if health records are not available.

Potential participants will be recruited from three selected KCCA clinics. Eligible AGYW will be identified through clinic records and service points, particularly LARC and HIV prevention programs. Community-based outreach will complement clinic recruitment, leveraging peer networks, community health workers, and targeted informational sessions to engage AGYW with diverse experiences. A peer-led snowball sampling approach will facilitate recruitment by encouraging initial participants to refer peers from their social networks. Recruitment will also extend to informal settlements and venues associated with sex work, such as bars and lodges, where AGYW are particularly vulnerable. Collaborations with venue managers and health centers will enable outreach to this population, ensuring equitable access to LARC and LAI-PrEP. Community sensitization events will educate AGYW on reproductive health, HIV prevention, and LARC and LAI-PrEP services. Study staff will identify and recruit KIs. The recruitment phase is expected to span approximately 4-6 months. A summary of study components, target populations, and methodologies is presented in Table 1.

**Table 1:** COMPASS study components, populations, methods, and eligibility criteria.

Study component	Target population	Method and data collection tool	Key measures and focus areas	Eligibility
Quantitative survey	AGYW^a^ aged 15-24 years attending selected KCCA^b^ health facilities	Structured questionnaire	Demographics, reproductive health history, preferences and willingness to use LARC^c^ and LAI-PrEP^d^, awareness and attitudes, perceived barriers and facilitators	Aged 15-24 years at enrolment. HIV-negative by rapid testing per national algorithm. Able and willing to provide written informed consent (parental waiver approved). Current use of LARC for ≥1 month or interest in LARC and PrEP or LAI-PrEP. Willing to comply with study procedures.
FGDs^e^	Subset of surveyed AGYW	Semistructured discussion guide	In-depth exploration of motivations, perceptions, social influences, and contextual factors affecting uptake and adherence to LARC and LAI-PrEP	Participated in quantitative survey component
IDIs^f^	Key stakeholders: health care providers, community leaders, peer educators, and program implementers	Interview guide	Perceptions of integrated service delivery, operational challenges, community norms, provider readiness, and system-level facilitators and barriers	Aged ≥18 years (see Study Population section for key informant details)

^a^AGYW: adolescent girls and young women.

^b^KCCA: Kampala Capital City Authority.

^c^LARC: long-acting reversible contraception.

^d^LAI-PrEP: long-acting injectable preexposure prophylaxis.

^e^FGD: focus group discussion.

^f^IDI: in-depth interview.

### Sample Size and Sampling Procedures

The quantitative sample size of up to 220 AGYW was estimated using the Kish-Leslie formula [51], assuming an 85% acceptability of LARC and LAI-PrEP if these services were made available. This sample size enables robust estimation of preferences, attitudes, and willingness to use integrated services, while accounting for variability in uptake across the study population. Participants will be purposively selected from the three KCCA clinics to ensure representation across urban, suburban, and peri-urban settings. For FGDs, a subset of 30 AGYW who completed the quantitative survey will be selected using stratified purposive sampling to ensure diversity in age, LARC and PrEP experience, and socio-demographic characteristics. This approach will enable in-depth exploration of factors influencing uptake and adherence. KIs will also be purposively sampled to capture a wide range of perspectives, professional roles, and influence over reproductive health and HIV prevention services. This combined sampling approach ensures a comprehensive understanding of both individual-level experiences and community-level factors affecting the integration and uptake of LARC and LAI-PrEP among AGYW in Uganda.

### Study Procedures

AGYW who express interest and meet the eligibility criteria will be consecutively enrolled at the designated health centers, enrolment and recruitment sites, which serve as key points for accessing reproductive health and HIV prevention services. Potential participants referred through community sensitization activities will first receive information about LARC and LAI-PrEP. Those expressing interest and meeting initial eligibility criteria will then undergo a screening process to confirm eligibility. Eligible participants will be taken through the informed consent process by trained study staff and, upon enrolment, assigned a unique study identifier to maintain confidentiality. Demographic information and quantitative data will be collected using structured paper-based questionnaires administered to AGYW.

We will endeavor to include AGYW aged 15-19 years and 20-24 years at a minimum ratio of 1:1.5 to ensure balanced age representation. We will also ensure diverse representation across socioeconomic statuses and educational backgrounds, providing a comprehensive understanding of preferences and perspectives across subpopulations.

**Figure 2:**
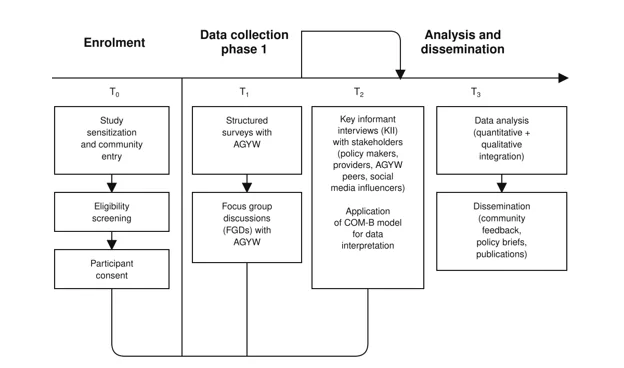
SPIRIT (Standard Protocol Items: Recommendations for Interventional Trials)-style schematic of the COMPASS Study. The figure summarizes the sequence of study activities, including community entry, participant recruitment and enrollment, quantitative and qualitative data collection, data analysis, interpretation of findings, and dissemination of study results. Legend: T0 – Community entry, participant recruitment & enrolment; T1 – Data collection; T2 – Data collection and interpretation; T3 – Data analysis & dissemination. This figure was generated using an artificial intelligence tool (OpenAI DALL·E). Prompt used: “Create a simple SPIRIT-style schematic illustrating study flow for the COMPASS Study from community entry to dissemination.” Source link to the generated image [55]. AGYW: adolescent girls and young women; COM-B: Capability, Opportunity, Motivation-Behavior; FGD: focus group discussion; KII: key informant interview.

Following the quantitative assessments, FGDs will be conducted to allow for a more in-depth exploration of key themes. FGDs will take place at the KCCA clinics to ensure accessibility and familiarity for participants. Sessions will be conducted in either English or Luganda, depending on participants’ language preferences, and will last approximately 90 minutes. Three FGDs will be held with AGYW, stratified by age groups (15-17, 18-20, and 21-24 years), with each participant completing a single FGD with others in the same age group. FGD guides will undergo pilot testing with a subset of the target population to ensure clarity, cultural appropriateness, and relevance. Before completing the FGD, participants will receive an introduction to LARC and LAI-PrEP as outlined in the FGD Guide. FGDs will be co-facilitated by trained AGYW peers to foster relatability, inclusivity, and alignment with Community-Based Participatory Research principles [52,53]. IDIs will be conducted among KIs enrolled in the study. Each key informant will complete a single IDI. Prior to the interview, participants will receive an introduction to LARC and LAI-PrEP, as outlined in the IDI Guide. Interviews will be conducted at participants’ workplaces to prioritize convenience and comfort, in either English or Luganda, depending on language preference, and will last approximately 60 minutes. The overall flow of study activities is summarized in SPIRIT (Standard Protocol Items: Recommendations for Interventional Trials) style schematic (Figure 2), which outlines the sequence of activities from community entry and participant enrolment through data collection, analysis, and dissemination [54].

### Data Collection

Quantitative and qualitative data will be collected sequentially to capture individual-level, social, and structural factors influencing integrated LARC and LAI-PrEP service delivery. Acceptability of integrated services, our primary outcome, will be measured using structured questionnaires that include Likert-scale items assessing willingness to use, perceived suitability, and compatibility with existing norms and values. The questionnaire was developed using the COM-B model and the BCW [48,49] and includes domains assessing health behaviors and preferences, interest and intent to use LARC and LAI-PrEP, perceived acceptability, barriers and facilitators, likelihood to recommend, and behavioral readiness. Perceived acceptability will be assessed using three four-point Likert-scale items evaluating whether (1) LARC fits within participants’ personal, cultural, and social context; (2) participants feel comfortable using LAI-PrEP within the community; and (3) perceived social acceptability among peers. Responses will be scored from 1 (Strongly Disagree) to 4 (Strongly Agree), with higher scores indicating greater acceptability. A composite acceptability score will be calculated by summing responses across these items. For descriptive analyses, acceptability scores will also be categorized into higher and lower acceptability using the observed median composite score of the study as the threshold. Other questionnaire domains will be analyzed individually to characterize interest, preferences, behavioral readiness, and perceived barriers and facilitators to integrated LARC and LAI-PrEP services. The complete survey questionnaire has been provided in Multimedia Appendix 3. Structured questionnaires, discussion guides, and interview tools are designed to assess sociodemographic characteristics, reproductive health history, HIV prevention behaviors, knowledge, attitudes, and perceived barriers and facilitators. Table 2 summarizes the key study domains, data sources, and participant groups.

**Table 2:** COMPASS study measures, data collection methods, and participant groups.

Domain or measure	Description or examples	Data source and method	Participants
Sociodemographic factors	Age, education, marital status, residence, employment status	Structured questionnaire	AGYW^a^
Reproductive and sexual health history	Parity, contraceptive use, fertility intentions, pregnancy history	Structured questionnaire	AGYW
HIV prevention behaviors	Partner communication about HIV prevention (PrEP)^b^, sexual debut, number of sexual partners, partner status, condom use and condomless sex, drug use before sex, recent STI^c^ diagnosis	Structured questionnaire	AGYW
Knowledge, attitudes, and perceptions	Understanding, acceptability, and preferences for LARC^d^ and LAI-PrEP^e^	Structured questionnaire; FGDs^f^	AGYW
Barriers and facilitators	Accessibility, cost, stigma, peer and partner influence, community norms	FGDs; IDIs^g^	AGYW, policymakers, health care providers, community leaders
Implementation context	Facility readiness, provider training, policy environment, supply chain	IDIs	Policymakers, providers
Behavior change strategies	Recommended messaging, delivery approaches, and community engagement preferences	FGDs; IDIs	AGYW, stakeholders

^a^AGYW: adolescent girls and young women.

^b^PrEP: preexposure prophylaxis.

^c^STI: sexually transmitted infection.

^d^LARC: long-acting reversible contraception.

^e^LAI-PrEP: long-acting injectable preexposure prophylaxis.

^f^FGD: focus group discussion.

^g^IDI: in-depth interviews.

### Data Analysis

Data analysis for this study will integrate both quantitative and qualitative approaches to provide a comprehensive understanding of AGYW engagement with LAI-PrEP and LARC. Quantitative data collection will involve administering structured questionnaires designed to capture preferences, attitudes, and perceptions regarding LAI-PrEP and LARC. The questionnaires will include Likert scale items to assess levels of agreement or disagreement with statements on acceptability, interest, and intent to use these interventions, alongside questions addressing barriers and facilitators to uptake. Additional sections will gather demographic information, reproductive health history, and HIV prevention behaviors. All data will be entered into REDCap [56] and exported to Stata for analysis. Analysis will begin with descriptive statistics to summarize participant characteristics and key variables, including acceptability, likelihood to recommend, and behavioral readiness. Frequencies, percentages, means, medians, standard deviations, and interquartile ranges will be reported to describe categorical and continuous data. Inferential analyses will then be conducted to identify associations and predictors. Correlations using Pearson or Spearman tests will examine relationships between continuous variables, such as age, education level, and preferences for LARC and LAI-PrEP. Likert scale responses will be collapsed into dichotomous outcomes (eg, agree vs disagree) and analyzed using chi-square tests to evaluate associations with demographic or behavioral factors.

Logistic regression models will be used to identify predictors of high acceptability and willingness to use integrated LARC and LAI-PrEP services. Variables considered clinically or theoretically relevant, together with those demonstrating an association at the bivariate analysis stage (*P*<.20), will be considered for inclusion in the multivariable models. Potential confounders, including age, education, socioeconomic status, marital status, prior contraceptive use, and HIV prevention history, will be retained in the final models where appropriate, based on subject-matter knowledge and statistical significance. Multicollinearity among independent variables will be assessed using variance inflation factors (VIFs) and correlation matrices. Where scientifically justified, interaction effects (for example, between age group and prior contraceptive use) will be explored by including interaction terms in the regression models. Model fit and assumptions will be evaluated using appropriate diagnostic procedures, including goodness-of-fit tests and assessment of influential observations. Adjusted odds ratios (aORs) with 95% CIs will be reported, and statistical significance will be assessed at a 2-sided α level of .05. Comparative analyses using 2-tailed *t* tests or ANOVA will further explore differences in preferences and attitudes across subgroups, including younger (15-19 years) versus older (20-24 years) AGYW and rural versus urban participants. For clarity of interpretation, results will be presented in tables summarizing numerical data and key statistics, including odds ratios (ORs) and *P* values. They will be visually illustrated with bar charts, pie charts, and histograms. Clustered bar charts and box plots will be used to highlight subgroup differences and significant predictors. This combined descriptive, inferential, and visualization approach will provide a robust understanding of AGYW preferences and attitudes toward LARC and LAI-PrEP, identify key facilitators and barriers to their uptake, and reveal demographic and behavioral predictors of positive engagement with these prevention methods.

Qualitative data from FGDs and IDIs will be audio-recorded, transcribed verbatim, translated into English where necessary, and analyzed using reflexive thematic analysis following Braun and Clarke's six-phase approach: (1) familiarization with the data, (2) generating initial codes, (3) constructing themes, (4) reviewing and refining themes, (5) defining and naming themes, and (6) producing the final report [57,58]. Codes will be developed inductively from the data while being interpreted in relation to the study objectives and the COM-B model. The multidisciplinary research team will engage in reflexive discussions throughout the analytical process to consider how their disciplinary backgrounds, experiences, and assumptions may influence data interpretation. An audit trail documenting coding decisions, codebook development, and theme refinement will be maintained throughout the analysis. Credibility will be enhanced through investigator triangulation, with different researchers independently reviewing transcripts and discussing emerging themes to reach consensus. Dependability and confirmability will be strengthened through regular analytical meetings and documentation of methodological decisions, while transferability will be supported by providing rich descriptions of the study setting, participants, and context. To support systematic analysis, NVivo software (Lumivero) will be used to organize transcripts, codes, and categories, while advanced query tools will facilitate the identification of thematic connections and overlaps. Quality assurance will be maintained through double coding of transcripts by 2 independent analysts, with discrepancies resolved through consensus discussions and peer debriefs to enhance validity. Once themes are consolidated, barriers to LARC and LAI-PrEP uptake will be mapped onto the COM-B model, categorizing them into domains of capability, opportunity, and motivation. This mapping will provide a structured behavioral lens through which challenges can be understood. Finally, insights from this process will guide the study team in collaboration with a behavioral science expert and key stakeholders to develop behavior change strategies informed by the BCW, ensuring that the interventions proposed are evidence-based, contextually appropriate, and designed to support AGYW in adopting and sustaining use of LARC and LAI-PrEP services. Following separate quantitative and qualitative analyses, findings from both components will be integrated using a convergent mixed methods approach. Integration will occur during the interpretation phase through triangulation to identify areas of convergence, complementarity, and divergence across data sources. Quantitative and qualitative findings will be merged using data mapping techniques (eg, joint displays) to identify connections and compare and contrast findings [59,60]. This approach will provide a comprehensive understanding of the behavioral, social, and health system factors influencing the uptake and implementation of integrated LARC and LAI-PrEP services.

### Ethical Considerations

The study received ethical approval from the Joint Clinical Research Centre (JCRC-REC 2025-143). Administrative clearance was obtained from the KCCA (DPHE/KCCA/1302/01) and approval from the Uganda National Council for Science and Technology (HS6232ES). All participants will provide written informed consent before any study procedures are carried out. Consent is viewed as an ongoing process conducted through open dialogue between study staff and participants. For AGYW, the screening and enrolment consent also includes embedded consent for possible participation in FGDs, eliminating the need for additional signatures. KIs similarly provide informed consent for enrolment and participation in IDIs at the screening and enrolment visit. Minors under 18 years may participate in the study. The JCRC-REC granted a waiver of parental consent because the study posed less than minimal risk. Consenting staff will follow Makerere University–Johns Hopkins University Research Collaboration’s (MU-JHU’s) Standard Operating Procedures using institutional review board (IRB)–approved forms in both English and Luganda, which are color-coded by participant group. Comprehension will be evaluated with an assessment of understanding tool, enrolling only those who demonstrate sufficient understanding and consent voluntarily. Additional safeguards will be implemented for participants aged 15-17 years to ensure their safety and well-being throughout the study. Interviews and FGDs will be conducted in private settings to protect confidentiality and encourage open discussion of sensitive topics. Participants will be reminded that participation is voluntary and that they may decline to answer any question, pause, or withdraw from the study at any time without penalty or loss of services. Study staff will be trained to recognize and respond appropriately to signs of distress. Participants requiring additional support will be referred to appropriate psychosocial, counseling, or clinical services in accordance with established referral procedures. All study personnel will receive training in Human Subjects Protection and Good Clinical Practice. The study will ensure confidentiality by removing participant identifiers from data, locking name-ID logs and signed consents in secure locations, and storing electronic data on password-protected computers. Participant rights, including the right to withdraw voluntarily, will be upheld. The study is classified as minimal risk but may cause discomfort or anxiety when answering sensitive questions related to reproductive health or HIV prevention. Although measures are in place to safeguard information, there remains a minor risk of unintended disclosure that could lead to social harm or stigma. Trained study staff will offer support for any concerns participants may have.

Participants in this study will not receive direct benefits; however, their involvement will help improve access to and acceptance of integrated LARC and LAI-PrEP services for AGYW in Uganda. The study allows participants to voice their opinions and help shape the design of more responsive and inclusive reproductive health and HIV prevention programs. Findings will be shared according to Uganda National Council for Science and Technology (UNCST) policies [61], prioritizing transparency and equity in dissemination to relevant audiences. Preliminary findings will be submitted to the local research ethics committees prior to any public release. The study results will be shared with study participants and community representatives via dissemination meetings at study sites, using culturally appropriate methods aligned with Good Participatory Practice principles [62] to ensure understanding and interaction. After local dissemination, findings will be presented to stakeholders, including KCCA health officials and partners. The study team will create policy briefs highlighting key findings and recommendations for the integrated delivery of LARC and LAI-PrEP among AGYW in Uganda. Nationally, results will also be shared with the Uganda Ministry of Health and relevant agencies through technical working groups, workshops, and dialogues. Additionally, findings will be presented to the scientific community at conferences and in peer-reviewed publications.

## Results

The study was funded in February 2026. Ethical and regulatory approvals were obtained before study initiation. Study preparation, including site initiation, staff training, finalization of study instruments, and community sensitization, was completed before participant recruitment. Participant recruitment began in March 2026 and was completed in July 2026, at selected KCCA clinics and community settings. A total of 220 AGYW and 20 KIs were enrolled, and both quantitative and qualitative data collection were completed in July 2026. Data cleaning and quality assurance activities were ongoing, and quantitative and qualitative data analyses were planned following completion of data collection. No major protocol modifications have been made since study initiation. Study findings are anticipated to be submitted for publication in 2027, following completion of data analysis and manuscript preparation.

## Discussion

### Anticipated Findings

This mixed methods cross-sectional implementation science study addresses a global health priority in HIV prevention and reproductive health programming by exploring the integration of two long-acting biomedical interventions, LARC and LAI-PrEP, for AGYW in Uganda. While both interventions have demonstrated high efficacy and acceptability, their integration within routine service delivery remains limited, and evidence on how best to deliver them jointly to meet the needs of AGYW is scarce. The introduction of lenacapavir and other long-acting prevention agents further underscores the need to design context-appropriate models that support combination prevention options [63,64]. COMPASS responds to this need by generating behavioral and implementation evidence to guide integrated service delivery in real-world settings.

A key strength of this protocol lies in its deliberate approach to intervention design. Too often, biomedical innovations are introduced without sufficient consideration of behavioral, social, and contextual factors that shape adoption and sustained use [65]. Prior studies have shown that interventions grounded in these frameworks are more likely to be effective and feasible, particularly in HIV prevention and reproductive health settings [66-70]. By integrating theoretical frameworks, COMPASS systematically identifies determinants of behavior and guides the development of tailored, theory-driven strategies. Being intentional in this design process ensures that emerging interventions are not only evidence-based but also relevant, feasible, and scalable within the Ugandan health system. Such deliberateness in the early design stages is particularly valuable, given the rapid evolution of long-acting HIV prevention technologies and the ongoing need to align service models with end-user preferences and health system realities.

The study’s participatory and co-design components further strengthen its methodological rigor. By engaging AGYW, peers, and health care providers throughout, the study captures diverse perspectives and enhances ownership of proposed solutions. COMPASS is also designed to empower AGYW, promoting autonomy, informed decision-making, and self-efficacy. Previous studies have shown that co-designing tools and interventions with end-users has been shown to improve the acceptability, feasibility, and sustainability of HIV prevention and reproductive health interventions among young populations [69,71,72]. The mixed methods design, which integrates quantitative surveys, qualitative interviews, and participatory co-design activities, allows triangulation of findings, providing a rich and tailored understanding of the social, behavioral, and structural factors that influence uptake of integrated LARC and LAI-PrEP services. This approach is expected to yield actionable strategies that can be tested in future implementation trials or scaled within existing programs.

We also recognized some limitations. The study’s focus on urban, suburban, and periurban settings in Kampala may limit the transferability of findings to rural areas, where health system capacity, cultural norms, and service delivery models may differ. Nevertheless, the inclusion of urban, peri-urban, and suburban clinics serving diverse populations provides evidence that is likely to be applicable to similar service delivery settings elsewhere in Uganda and in other sub-Saharan African countries facing comparable HIV and reproductive health challenges among AGYW. Rather than aiming for external validity, this implementation science study seeks to generate relevant evidence that can inform the adaptation of integrated LARC and LAI-PrEP delivery strategies. Future implementation in rural settings should consider differences in health system capacity, access to youth-friendly services, cultural norms, and community engagement approaches when adapting the proposed strategies.

Participant recruitment may also be subject to selection bias because clinic-based recruitment may preferentially enroll AGYW who are already engaged with health services, while peer-led snowball sampling may overrepresent individuals within existing social networks. To reduce this potential bias, the study incorporates community-based recruitment through peer networks, community health workers, community sensitization activities, and outreach beyond clinic settings, in addition to recruitment across three KCCA clinics that serve different populations: Kisenyi (urban), Kawaala (peri-urban), and Komamboga (suburban). Although these approaches are intended to broaden participant representation, some residual selection bias may remain.

Additionally, reliance on self-reported measures regarding sexual behavior, contraceptive use, and PrEP adherence may be affected by recall and social desirability bias [73-75]. Mitigation strategies, including confidential data collection methods and triangulation across multiple data sources, are built into the design to reduce these risks. Nonetheless, these limitations provide opportunities for future work, including follow-up studies to assess the applicability of identified strategies in rural contexts and longitudinal evaluations to determine their impact on sustained uptake and adherence. Future directions will also include pilot testing of co-designed interventions to refine their feasibility, cost-effectiveness, and scalability across diverse service delivery settings.

The evolving global HIV prevention funding and policy landscape further reinforces the significance of this research. Recent reductions and policy shifts in funding for the President’s Emergency Plan for AIDS Relief (PEPFAR) and the United States Agency for International Development (USAID) have heightened the emphasis on cost-efficiency, sustainability, and local ownership in HIV programming [76,77]. Although this study is not expected to be directly affected by these funding shifts, its focus on community-driven and contextually relevant implementation approaches aligns with global priorities to optimize resources and strengthen health systems.

In this environment, implementation science research becomes even more critical, as it provides evidence to guide the integration of new biomedical tools into existing infrastructure. Simultaneously, the increasing global enthusiasm for long-acting prevention agents, such as lenacapavir, highlighted by regulatory approvals and global guidelines, underscores the timeliness of COMPASS. The study’s findings will contribute practical evidence on how future long-acting products can be delivered effectively and equitably, particularly for AGYW who remain at elevated risk but are often underserved in current programs.

### Conclusions

This study has important future implications. It exemplifies a deliberate, theory-informed, and participatory approach to designing interventions for AGYW. The findings are expected to inform future implementation studies, intervention development, and policy discussions regarding integrated delivery of LARC and LAI-PrEP services for AGYW in Uganda and similar high HIV burden settings. Evidence from the participatory design process may guide the implementation of similar integrated interventions in other high-burden settings, fostering sustainable improvements in health outcomes for young women across sub-Saharan Africa. Beyond its immediate scope, this study provides a framework for designing and evaluating future long-acting prevention interventions, ensuring they are grounded in behavioral science, co-created with communities, and responsive to evolving global and funding priorities.

## Appendix

Multimedia Appendix 1 Survey questionnaire.

Multimedia Appendix 2 Focus group discussion guide.

Multimedia Appendix 3 In-depth interview guide.

## Abbreviations

AGYW: adolescent girls and young women
aOR: adjusted odds ratio
BCW: Behavior Change Wheel
COM-B: Capability, Opportunity, Motivation-Behavior
FGD: focus group discussion
IDI: in-depth interview
IRB: institutional review board
KI: key informant
KCCA: Kampala Capital City Authority
LAI-PrEP: long-acting injectable preexposure prophylaxis
LARC: long-acting reversible contraception
MU-JHU: Makerere University–Johns Hopkins University Research Collaboration
OR: odds ratio
PEPFAR: President’s Emergency Plan for AIDS Relief
SPIRIT: Standard Protocol Items: Recommendations for Interventional Trials
SRH: sexual and reproductive health
UNAIDS: Joint United Nations Programme on HIV and AIDS
UNCST: Uganda National Council for Science and Technology
USAID: United States Agency for International Development
VIF: variance inflation factor
WHO: World Health Organization

## Data Availability

The data shared will not disclose personally identifiable information, and its intended use will comply with other regulatory requirements and good research practice; and will not in any way infringe on the rights of the study participants or otherwise affect them adversely. The data recipient will agree not to forward data to nonpermitted parties and will agree to comply with the data sharing policy. Access to databases generated under the study will be available for educational, research, and nonprofit purposes as approved by the primary Institutional Review Board.

## References

[1] Pettifor A, Stoner Marie, Pike Carey, Bekker Linda-Gail (2018). Adolescent lives matter: preventing HIV in adolescents. Curr Opin HIV AIDS.

[2] (2025). World Fertility 2024. United Nations Organization.

[3] (2025). Fertility rate, total (births per woman) - Uganda. World Bank Group.

[4] Namukisa M, Kamacooko O, Lunkuse JF, Ruzagira E, Price MA, Mayanja Y (2023). Incidence of unintended pregnancy and associated factors among adolescent girls and young women at risk of HIV infection in Kampala, Uganda. Front Reprod Health.

[5] Ayalew HG, Liyew AM, Tessema ZT, Worku MG, Tesema GA, Alamneh TS, Teshale AB, Yeshaw Y, Alem AZ (2022). Prevalence and factors associated with unintended pregnancy among adolescent girls and young women in sub-Saharan Africa, a multilevel analysis. BMC Womens Health.

[6] (2022). Uganda demographic and health survey 2022 main report. Uganda Bureau of Statistics.

[7] Global AIDS strategy 2021-2026 — end inequalities. End AIDS. UNAIDS, Global AIDS Update.

[8] Obeagu EI, Obeagu GU (2024). Preventive measures against HIV among Uganda's youth: Strategies, implementation, and effectiveness. Medicine (Baltimore).

[9] (2020). The Uganda HIV/AIDS Country Progress Report 2020. Uganda AIDS Commission.

[10] (2022). Consolidated guidelines on HIV prevention, testing, treatment, service delivery and monitoring: recommendations for a public health approach. World Health Organization.

[11] Joint United Nations Programme on HIV/AIDS (UNAIDS), and M.o.H. (Uganda), Uganda HIV/AIDS fact sheet 2025. Uganda AIDS Commission.

[12] Karim SSA, Baxter C (2019). HIV incidence rates in adolescent girls and young women in sub-Saharan Africa. The Lancet Global Health.

[13] Delany-Moretlwe S, Hughes JP, Bock P, Ouma SG, Hunidzarira P, Kalonji D, Kayange N, Makhema J, Mandima P, Mathew C, Spooner E, Mpendo J, Mukwekwerere P, Mgodi N, Ntege PN, Nair G, Nakabiito C, Nuwagaba-Biribonwoha H, Panchia R, Singh N, Siziba B, Farrior J, Rose S, Anderson PL, Eshleman SH, Marzinke MA, Hendrix CW, Beigel-Orme S, Hosek S, Tolley E, Sista N, Adeyeye A, Rooney JF, Rinehart A, Spreen WR, Smith K, Hanscom B, Cohen MS, Hosseinipour MC (2022). Cabotegravir for the prevention of HIV-1 in women: results from HPTN 084, a phase 3, randomised clinical trial. Lancet.

[14] Engelbert Bain L, Amu H, Enowbeyang Tarkang E (2021). Barriers and motivators of contraceptive use among young people in Sub-Saharan Africa: A systematic review of qualitative studies. PLoS One.

[15] Celum CL, Delany‐Moretlwe S, McConnell M, van Rooyen H, Bekker L, Kurth A, Bukusi E, Desmond C, Morton J, Baeten JM (2015). Rethinking HIV prevention to prepare for oral PrEP implementation for young African women. Journal of the International AIDS Society.

[16] Celum CL, Delany-Moretlwe Sinead, Baeten Jared M, van der Straten Ariane, Hosek Sybil, Bukusi Elizabeth A, McConnell Margaret, Barnabas Ruanne V, Bekker Linda-Gail (2019). HIV pre-exposure prophylaxis for adolescent girls and young women in Africa: from efficacy trials to delivery. J Int AIDS Soc.

[17] Nalwadda G, Mirembe F, Byamugisha J, Faxelid E (2010). Persistent high fertility in Uganda: young people recount obstacles and enabling factors to use of contraceptives. BMC Public Health.

[18] Ameyaw EK, Budu E, Sambah F, Baatiema L, Appiah F, Seidu A, Ahinkorah BO (2019). Prevalence and determinants of unintended pregnancy in sub-Saharan Africa: A multi-country analysis of demographic and health surveys. PLoS One.

[19] Kriel Y, Milford C, Cordero JP, Suleman F, Steyn PS, Smit JA (2023). A continuum of individual-level factors that influence modern contraceptive uptake and use: perspectives from community members and healthcare providers in Durban, South Africa. Contracept Reprod Med.

[20] Idris IB, Syed Soffian SS, Baharom M, Baharuddin UM, Hashim S, Nawi AM (2022). Influence of sociocultural beliefs and practices on contraception: a systematic review. Women Health.

[21] Ajayi AI, Ezegbe HC (2020). Association between sexual violence and unintended pregnancy among adolescent girls and young women in South Africa. BMC Public Health.

[22] Ninsiima LR, Chiumia IK, Ndejjo R (2021). Factors influencing access to and utilisation of youth-friendly sexual and reproductive health services in sub-Saharan Africa: a systematic review. Reprod Health.

[23] Menon S, COMMITTEE ON ADOLESCENCE (2020). Long-acting reversible contraception: specific issues for adolescents. Pediatrics.

[24] Ott MA, Sucato GS, Committee on Adolescence (2014). Contraception for adolescents. Pediatrics.

[25] American College of Obstetricians and Gynecologists (2018). ACOG committee opinion no. 735: adolescents and long-acting reversible contraception: implants and intrauterine devices. Obstet Gynecol.

[26] Hillard PJ (2013). Practical tips for intrauterine devices use in adolescents. J Adolesc Health.

[27] Sambah F, Aboagye RG, Seidu A, Tengan CL, Salihu T, Ahinkorah BO (2022). Long-acting reversible contraceptives use among adolescent girls and young women in high fertility countries in sub-Saharan Africa. Reprod Health.

[28] Kungu WA, Khasakhala A, Agwanda A (2020). Use of long-acting reversible contraception among adolescents and young women in Kenya. PLoS One.

[29] Traynor Kate (2025). CDTM services improve LAI-PrEP management for highly vulnerable patients. Am J Health Syst Pharm.

[30] (2021). Integration of HIV testing and linkage in family planning and contraception services: implementation brief. World Health Organization.

[31] Myers JT, Ellman TM, Westhoff C (2015). Injectable agents for pre-exposure prophylaxis: lessons learned from contraception to inform HIV prevention. Curr Opin HIV AIDS.

[32] Patel P, Sato Kimi, Bhandari Neeta, Kanagasabai Udhayashankar, Schelar Erin, Cooney Caroline, Eakle Robyn, Klucking Sara, Toiv Nora, Saul Janet, PEPFAR PrEP stakeholders∗ (2022). From policy to practice: uptake of pre-exposure prophylaxis among adolescent girls and young women in United States President's Emergency Plan for AIDS Relief-supported countries, 2017-2020. AIDS.

[33] Dovel K, Shaba F, Offorjebe OA, Balakasi K, Nyirenda M, Bell TG, Havlir DV Long-acting injectable PrEP. National Alliance of State & Territorial AIDS Directors (NASTAD).

[34] Ortblad KF, Kuo AP, Reedy AM, Johnson CC, Ngure K, Wagner AD, Franke MF (2022). Interventions to increase the uptake and continuation of pre-exposure prophylaxis (PrEP) by adolescent girls and young women at high risk of HIV in low-income and middle-income countries: a scoping review. BMJ Glob Health.

[35] Gotsche CI, Steyn Petrus S, Narasimhan Manjulaa, Rodolph Michelle, Baggaley Rachel, Kiarie James N (2023). Integrating pre-exposure prophylaxis of HIV infection into family planning services: a scoping review. BMJ Sex Reprod Health.

[36] van Vliet MM, Hendrickson C, Nichols BE, Boucher CA, Peters RP, van de Vijver DA (2019). Epidemiological impact and cost-effectiveness of providing long-acting pre-exposure prophylaxis to injectable contraceptive users for HIV prevention in South Africa: a modelling study. J Int AIDS Soc.

[37] Mugwanya KK, Kinuthia J (2021). Integrating Pre-Exposure Prophylaxis Delivery in Public Health Family Planning Clinics: Lessons Learned From a Programmatic Implementation Project in Kenya. Front Reprod Health.

[38] (2020). OPTIONS integrated service delivery: findings on integration of oral PrEP into family planning services for adolescent girls and young women in sub-Saharan Africa. OPTIONS Consortium.

[39] Martin CE, Muhwava LS, Dada S, Scorgie F, Mullick S (2025). Experiences with Oral Pre-Exposure Prophylaxis (PrEP) Service Delivery and Use Among Adolescent Girls and Young Women (AGYW) in Routine Primary Care Settings, South Africa. AIDS Behav.

[40] Barnabee G, Billah I, Ndeikemona L, Silas L, Ensminger A, MacLachlan E, Korn AK, Mawire S, Fischer-Walker C, Ashipala L, Forster N, O'Malley G, Velloza J (2023). PrEP uptake and early persistence among adolescent girls and young women receiving services via community and hybrid community-clinic models in Namibia. PLoS One.

[41] Butler V, Kutywayo A, Martin CE, Pleaner M, Mojapele MV, Ncube S, Fipaza Z, Mundeta B, Mullick S (2023). Implementing Differentiated and Integrated HIV Prevention Services for Adolescent Girls and Young Women: Experiences From Oral PrEP Rollout in Primary Care Services in South Africa. J Adolesc Health.

[42] Bader B, Coenen M, Hummel J, Schoenweger P, Voss S, Jung-Sievers C (2023). Evaluation of community-based health promotion interventions in children and adolescents in high-income countries: a scoping review on strategies and methods used. BMC Public Health.

[43] Kakande ER, Ayieko J, Sunday H, Biira E, Nyabuti M, Agengo G, Kabami J, Aoko C, Atuhaire HN, Sang N, Owaranganise A, Litunya J, Mugoma EW, Chamie G, Peng J, Schrom J, Bacon MC, Kamya MR, Havlir DV, Petersen ML, Balzer LB, SEARCH Study Team (2023). A community-based dynamic choice model for HIV prevention improves PrEP and PEP coverage in rural Uganda and Kenya: a cluster randomized trial. J Int AIDS Soc.

[44] Kabwama SN, Kiwanuka SN, Mapatano MA, Fawole OI, Seck I, Namale A, Ndejjo R, Kizito S, Monje F, Bosonkie M, Egbende L, Bello S, Bamgboye EA, Dairo MD, Adebowale AS, Salawu MM, Afolabi RF, Diallo I, Leye MMM, Ndiaye Y, Fall M, Bassoum O, Alfvén T, Sambisa W, Wanyenze RK (2022). Private sector engagement in the COVID-19 response: experiences and lessons from the Democratic Republic of Congo, Nigeria, Senegal and Uganda. Global Health.

[45] Vandormael A, Akullian A, Siedner M, de Oliveira T, Bärnighausen Till, Tanser F (2019). Declines in HIV incidence among men and women in a South African population-based cohort. Nat Commun.

[46] (2021). Building health systems resilience for universal health coverage and health security during the COVID-19 pandemic and beyond: WHO position paper. World Health Organization.

[47] (2021). Understanding measures of progress towards the 95-95-95 HIV testing, treatment and viral suppression targets. UNAIDS.

[48] Pilat D (2021). The COM-B model for behavior change. The Decision Lab.

[49] Michie S, van Stralen MM, West R (2011). The behaviour change wheel: a new method for characterising and designing behaviour change interventions. Implement Sci.

[50] (2025). COM-B model, adapted for the COMPASS Study.

[51] Lavoie MC, Okui L, Blanco N, Stoebenau K, Magidson JF, Gokatweng G, Ikgopoleng K, Charurat ME, Ndwapi N (2023). Feasibility and acceptability of peer-delivered interventions using mHealth for PrEP services among adolescent girls and young women in DREAMS program in Botswana. Glob Health Action.

[52] Collins SE, Clifasefi SL, Stanton J, Straits KJE, Gil-Kashiwabara E, Rodriguez Espinosa P, Nicasio AV, Andrasik MP, Hawes SM, Miller KA, Nelson LA, Orfaly VE, Duran BM, Wallerstein N, The LEAP Advisory Board (2018). Community-based participatory research (CBPR): towards equitable involvement of community in psychology research. American Psychologist.

[53] Hacker K (2013). Community-Based Participatory Research.

[54] Chan AW, Tetzlaff JM, Altman DG, Laupacis A, Gøtzsche Peter C, Krleža-Jerić Karmela, Hróbjartsson Asbjørn, Mann H, Dickersin K, Berlin JA, Doré Caroline J, Parulekar WR, Summerskill WSM, Groves T, Schulz KF, Sox HC, Rockhold FW, Rennie D, Moher D (2013). SPIRIT 2013 statement: defining standard protocol items for clinical trials. Ann Intern Med.

[55] (2025). AI-generated figure for the COMPASS Study: SPIRIT-style schematic. OpenAI.

[56] Odukoya O, Nenrot D, Adelabu H, Katam N, Christian E, Holl J, Okonkwo A, Kocherginsky M, Kim K, Akanmu S, Abdulkareem FB, Anorlu R, Musa J, Lesi O, Hawkins C, Okeke O, Adeyemo WL, Sagay S, Murphy R, Hou L, Ogunsola FT, Wehbe FH (2021). Application of the research electronic data capture (REDCap) system in a low- and middle income country- experiences, lessons, and challenges. Health Technol (Berl).

[57] Braun V, Clarke V (2023). Toward good practice in thematic analysis: Avoiding common problems and be(com)ing a researcher. Int J Transgend Health.

[58] Braun V, Clarke V (2023). Toward good practice in thematic analysis: avoiding common problems and be(com)ing a knowing researcher. Int J Transgend Health.

[59] Fetters MD, Tajima C (2022). Joint displays of integrated data collection in mixed methods research. International Journal of Qualitative Methods.

[60] Guetterman TC, Fetters MD, Creswell JW (2015). Integrating quantitative and qualitative results in health science mixed methods research through joint displays. Ann Fam Med.

[61] (2025). Uganda National Council for Science and Technology Open science policy.

[62] AVAC U (2025). Good participatory practice: guidelines for biomedical HIV prevention trials (draft second edition).

[63] Bekker LG, Das M, Abdool Karim Q, Ahmed K, Batting J, Brumskine W, Gill K, Harkoo I, Jaggernath M, Kigozi G, Kiwanuka N, Kotze P, Lebina L, Louw CE, Malahleha M, Manentsa M, Mansoor LE, Moodley D, Naicker V, Naidoo L, Naidoo M, Nair G, Ndlovu N, Palanee-Phillips T, Panchia R, Pillay S, Potloane D, Selepe P, Singh N, Singh Y, Spooner E, Ward AM, Zwane Z, Ebrahimi R, Zhao Y, Kintu A, Deaton C, Carter CC, Baeten JM, Matovu Kiweewa F, PURPOSE 1 Study Team (2024). Twice-Yearly Lenacapavir or Daily F/TAF for HIV Prevention in Cisgender Women. N Engl J Med.

[64] (2025). Guidelines on lenacapavir for HIV prevention and testing strategies for long-acting injectable pre-exposure prophylaxis.

[65] (2023). The Innovation Life Cycle in Health and Medicine and the Challenge of Equity, in Toward Equitable Innovation in Health and Medicine: A Framework.

[66] Denison JA, Willis K, DeLong SM, Sievwright KM, Agwu AL, Arrington-Sanders R, Kaufman MR, Prabhu S, Williams AM, Fields EL, Alexander KA, Lee L, Yang C, Johns Hopkins University Center for AIDS Research AdolescentYoung Adult Scientific Working Group (2024). Advancing Adolescent and Young Adult HIV Prevention and Care and Treatment Through Use of Multi-level Theories and Frameworks: A Scoping Review and Adapted HIV Ecological Framework. AIDS Behav.

[67] Murphy K, Berk J, Muhwava-Mbabala L, Booley S, Harbron J, Ware L, Norris S, Zarowsky C, Lambert EV, Levitt NS (2023). Using the COM-B model and Behaviour Change Wheel to develop a theory and evidence-based intervention for women with gestational diabetes (IINDIAGO). BMC Public Health.

[68] Bennett RJ, Bucks RS, Saulsman L, Pachana NA, Eikelboom RH, Meyer CJ (2023). Use of the behaviour change wheel to design an intervention to improve the provision of mental wellbeing support within the audiology setting. Implement Sci Commun.

[69] Flanagan S, Gorstein A, Nicholson M, Bradish S, Amanyire D, Gidudu A, Aucur F, Twesigye J, Kyateka F, Balamaga S, Buttenheim A, Zimmerman E (2021). Behavioural intervention for adolescent uptake of family planning: a randomized controlled trial, Uganda. Bull World Health Organ.

[70] Diamond-Smith N, Walker D, Afulani PA, Donnay F, Lin S(Y, Peca E, Stanton ME (2023). The case for using a behavior change model to design interventions to promote respectful maternal care. Glob Health Sci Pract.

[71] (2024). A Systematic Review on Adolescent Contraceptive Usage.

[72] Burchett HED (2022). Which structural interventions for adolescent contraceptive use have been evaluated in low- and middle-income countries. Int J Environ Res Public Health.

[73] King BM (2022). The influence of social desirability on sexual behavior surveys: a review. Archives of Sexual Behavior.

[74] Nakiganda LJ, Grulich AE, Poynten IM, Serwadda D, Bazaale JM, Jin J, Bavinton BR (2022). Self-reported and pill count measures of adherence to oral HIV PrEP among female sex workers living in South-Western Uganda. PLoS One.

[75] Stuart GS, Grimes DA (2009). Social desirability bias in family planning studies: a neglected problem. Contraception.

[76] Finzi DN (2024). Breakthrough Lenacapavir Trial Builds on Decades of NIH Investment in Basic Science. National Institutes of Health.

[77] Hontelez JA, Goymann H, Berhane Y, Bhattacharjee P, Bor J, Chabata ST, Cowan F, Kimani J, Knox J, Lora WS, Lungu C, Manne-Goehler J, Mauti J, Moshabela M, Mpembeni RM, Wa Mwanza M, Ndung'u T, Omondi E, Phiri S, Siedner M, Tanser FC, de Vlas SJ, Bärnighausen TW (2025). The impact of the PEPFAR funding freeze on HIV deaths and infections: a mathematical modelling study of seven countries in sub-saharan Africa. EClinicalMedicine.

