# Supplementary material for: Community Perspectives and Behavior Change Strategies for Integrated Long-Acting Reversible Contraception and Long-Acting Injectable Preexposure Prophylaxis Roll Out for Adolescent Girls and Young Women in Uganda (COMPASS): Protocol for a Mixed Methods Implementation Science Study
Source: JMIR Res Protoc. 2026 Sep 11;15:e88635. doi: 10.2196/88635 (PMC13615377; doi:10.2196/88635)
Supplement: Supplementary file 1 [file resprot_v15i1e88635_app1.docx]

**Survey Questionnaire**

**Study: COMPASS: Community Perspectives and Behavioral Change Strategies on LARC and LAI-PrEP Rollout Among AGYW in Uganda**

Questionnaire for AGYW

**Confidentiality Statement: Long-acting reversible contraceptives (LARC) are effective birth control options that can last for several years and can be removed whenever needed. Long-acting injectable PrEP (LAI-PrEP) is an antiretroviral medication administered every two to six months and is highly effective in preventing HIV infection.**

This questionnaire gathers data based on the COM-B model (Capability, Opportunity, Motivation) while addressing the specified aspects: interest and intent, perceived acceptability, likelihood to recommend, behavioral readiness, health behaviors, preferences, and barriers/facilitators to LARC and LAI-PrEP use. Your responses are confidential and will only be used for research purposes.

**Section 1: Demographics, Reproductive Health, and HIV Risk Factors**

1. What is your age?
   - ☐ 15 years	☐ 16 years	☐ 17 years
   - ☐ 18 years	☐ 19 years	☐ 20 years
   - ☐ 21 years	☐ 22 years	☐ 23 years	☐ 24 years
2. What is your highest level of education?
   - ☐ No formal education
   - ☐ Primary school
   - ☐ Secondary school
   - ☐ Tertiary/University
3. What is your current marital status?
   - ☐ Single
   - ☐ Married
   - ☐ Divorced/Separated
   - ☐ Widowed
4. What is your current employment status?
   - ☐ Student
   - ☐ Employed
   - ☐ Unemployed
   - ☐ Self-employed
5. Do you currently use any contraceptive method?
   - ☐ Yes (Specify: ____________________________)
   - ☐ No
6. Have you ever used a long-acting reversible contraceptive (LARC) method (e.g., implant, Depo Provera or intrauterine device)?

- ☐ Yes
- ☐ No

7. What is your main source of information about family planning?

- ☐ Mass media (radio, television, internet)
- ☐ Family or friends
- ☐ Health facility/health workers
- ☐ Community health workers
- ☐ Other (Specify: ______________________)

8. How many living children do you currently have?

- Number: __________________

9. Would you like to have more children in the future?

- ☐ Yes
- ☐ No

10. Have you discussed family planning or contraceptive use with your current or most recent partner?

- ☐ Yes
- ☐ No
- ☐ Not applicable (I have never had a partner)

11. If you wanted to use a family planning method, how supportive do you think your partner would be?

- ☐ Very supportive
- ☐ Somewhat supportive
- ☐ Not supportive
- ☐ I do not know
- ☐ Not applicable (No partner)

12. Who usually makes the decision about whether to use family planning in your relationship?

- ☐ Mostly myself
- ☐ Mostly my partner
- ☐ We decide together
- ☐ Someone else influences the decision (Specify: ____________________)
- ☐ Not applicable (No partner)

13. Would you feel comfortable discussing HIV prevention methods (such as PrEP) with your partner?

- ☐ Yes
- ☐ No
- ☐ Not sure
- ☐ Not applicable (No partner)

14. At what age did you first have sexual intercourse or begin living with a partner?

- Age: __________________

15. In the past one month, how many different partners have you had sexual intercourse with?

- Number: __________________

16. Are you currently living with your partner or spouse?

- ☐ Yes
- ☐ No
- ☐ Not applicable (No partner)

17. Do you usually use condoms during sexual intercourse?

- ☐ All the time/very frequently
- ☐ Sometimes
- ☐ Never

18. In the past three months, have you had sexual intercourse without using a condom?

- ☐ Yes
- ☐ No

19. In the past six months, have you used any illicit drugs before engaging in sexual intercourse?

- ☐ Yes
- ☐ No

20. In the past three months, have you been diagnosed with a sexually transmitted infection (STI) by a health professional?

- ☐ Yes
- ☐ No

21. How long does it take you to walk to the nearest health facility?

- ☐ Less than 30 minutes
- ☐ 30 minutes or more

**Section 2: Health Behaviors and Preferences for LARC and LAI-PrEP**

| **Statement** | **Strongly Agree** | **Agree** | **Disagree** | **Strongly Disagree** |
| --- | --- | --- | --- | --- |
| 22. I consistently use contraception to prevent pregnancy. | ☐ | ☐ | ☐ | ☐ |
| 23. I have used or considered using PrEP to prevent HIV. | ☐ | ☐ | ☐ | ☐ |
| 24. I prefer long-acting methods over daily-use methods for contraception or HIV prevention. | ☐ | ☐ | ☐ | ☐ |
| 25. I trust healthcare providers when making decisions about LARC and LAI-PrEP. | ☐ | ☐ | ☐ | ☐ |

26. If you use contraception, which method do you prefer?
    - ☐ Daily pill
    - ☐ Injectable
    - ☐ Implant
    - ☐ Intrauterine device (IUD)
    - ☐ None
    - ☐ Other (Specify: ______________________)
27. If you were to use PrEP, which option would you prefer?
    - ☐ Daily oral PrEP
    - ☐ Long-acting injectable PrEP
    - ☐ Other (Specify: ______________________)

**Section 3: Interest and Intent (Capability and Motivation)**

| **Statement** | **Strongly Interested** | **Somewhat Interested** | **Not Interested** | **Not Sure** |
| --- | --- | --- | --- | --- |
| 28. I am interested in using LARC if available. | ☐ | ☐ | ☐ | ☐ |
| 29. I am interested in using LAI-PrEP if available. | ☐ | ☐ | ☐ | ☐ |
| 30. I am willing to use both LARC and LAI-PrEP at the same time if both were available | ☐ | ☐ | ☐ | ☐ |

31. What would motivate you to use LARC or LAI-PrEP? (Select all that apply)

- ☐ Convenience
- ☐ Long-term protection
- ☐ Recommendation from a healthcare provider
- ☐ Peer recommendation
- ☐ Other (Specify: ________________________)

**Section 4: Perceived Acceptability (Opportunity and Motivation)**

| **Statement** | **Strongly Agree** | **Agree** | **Disagree** | **Strongly Disagree** |
| --- | --- | --- | --- | --- |
| 32. LARC fits well within my personal, cultural, and social context. | ☐ | ☐ | ☐ | ☐ |
| 33. I feel comfortable using LAI-PrEP in my community. | ☐ | ☐ | ☐ | ☐ |
| 34. I feel that using LARC or LAI-PrEP would be socially acceptable among my peers. | ☐ | ☐ | ☐ | ☐ |

35. What concerns, if any, would you have about using LARC or LAI-PrEP?

- ☐ Fear of side effects
- ☐ Stigma in the community
- ☐ Lack of privacy
- ☐ Cost
- ☐ Other (Specify: __________________________)

**Section 5: Barriers and Facilitators to LARC and LAI-PrEP Use**

| **Statement** | **Strongly Agree** | **Agree** | **Disagree** | **Strongly Disagree** |
| --- | --- | --- | --- | --- |
| 36. I have enough information to decide whether to use LARC or LAI-PrEP. | ☐ | ☐ | ☐ | ☐ |
| 37. I believe my family/partner would support my use of LARC or LAI-PrEP. | ☐ | ☐ | ☐ | ☐ |
| 38. Access to a trusted healthcare provider would increase my willingness to use LARC or LAI-PrEP. | ☐ | ☐ | ☐ | ☐ |
| 39. Fear of side effects would prevent me from using LARC or LAI-PrEP. | ☐ | ☐ | ☐ | ☐ |
| 40. I would prefer community-based access to LARC and LAI-PrEP services rather than clinic-based services. | ☐ | ☐ | ☐ | ☐ |
| 41. I would prefer to receive LARC and LAI-PrEP services during the same visit at the same location | ☐ | ☐ | ☐ | ☐ |

**Section 6: Likelihood to Recommend (Motivation)**

| **Statement** | **Strongly Agree** | **Agree** | **Disagree** | **Strongly Disagree** |
| --- | --- | --- | --- | --- |
| 42. I would recommend LARC to my peers. | ☐ | ☐ | ☐ | ☐ |
| 43. I would recommend LAI-PrEP to my peers. | ☐ | ☐ | ☐ | ☐ |
| 44. I would feel confident advocating for LARC and LAI-PrEP in my community. | ☐ | ☐ | ☐ | ☐ |

45. If no, what would prevent you from recommending LARC or LAI-PrEP to your peers?

- ☐ Lack of information
- ☐ Concerns about safety
- ☐ Community norms
- ☐ Other (Specify: __________________________)

**Section 7: Behavioral Readiness (Capability, Opportunity, Motivation)**

| **Statement** | **Strongly Agree** | **Agree** | **Disagree** | **Strongly Disagree** |
| --- | --- | --- | --- | --- |
| 46. I am likely to seek out more information about LARC and LAI-PrEP. | ☐ | ☐ | ☐ | ☐ |
| 47. If services were easily accessible, I would consider using LARC or LAI-PrEP. | ☐ | ☐ | ☐ | ☐ |
| 48. I would attend an informational session on LARC and LAI-PrEP. | ☐ | ☐ | ☐ | ☐ |

**End of Survey**

Thank you for your time and participation! Your responses will help improve services for AGYW in your community.
