# Supplementary material for: Community Perspectives and Behavior Change Strategies for Integrated Long-Acting Reversible Contraception and Long-Acting Injectable Preexposure Prophylaxis Roll Out for Adolescent Girls and Young Women in Uganda (COMPASS): Protocol for a Mixed Methods Implementation Science Study
Source: JMIR Res Protoc. 2026 Sep 11;15:e88635. doi: 10.2196/88635 (PMC13615377; doi:10.2196/88635)
Supplement: Supplementary file 2 [file resprot_v15i1e88635_app2.doc]

**Focus Group Discussion (FGD) Guide**

**Study: COMPASS: Community Perspectives and Behavioral Change Strategies on LARC and LAI-PrEP Rollout Among AGYW in Uganda**

FGD guide for selected Adolescent Girls and Young Women (AGYW)

**INSTRUCTIONS for the Facilitators: How to use the FGD Guide**

1. Section headings are noted with blue highlighting
2. Notes to the facilitators are in **[brackets]*.***
3. Text marked as ***Script*** is meant to be read aloud to participants. This text either explains a topic/section to the respondents or provides some information or instruction. The facilitator can then rephrase in their own words or clarify to the participant as necessary.
4. The FGD guide is set up as a table. The first column notes the guiding questions. Probes are indicated with bullets in the second column. If you find that the participants do not provide much information in response to the primary question, use these probes to encourage further discussion. Use the probes to make sure that all issues are addressed, but move on when you feel you are starting to hear repetitive information. Note that participants may provide responses to some questions, so some of the probes may not be needed.
5. The FGD guide is not meant to be used to take notes; please use a separate notes form.
6. To the extent possible, try to maintain a smooth conversational flow. It is up to the facilitator’s discretion if a question should be skipped if that topic has already been covered by responses to questions earlier in the discussion. Please ensure that by the end of the discussion, all the key topics/questions have been covered.

**[Note to facilitator: Turn on the Recorder. Begin recording now.]**

**Script:** *Before we start, can you confirm for the recorder that you have already provided written informed consent to take part in this discussion?*

**[Note to facilitator: Wait for oral confirmation to begin].**

*I want to remind you that what we discuss here will be kept confidential; we will not share your personal information or responses with anyone outside of the study. We will not associate your real name with anything you say in the focus group or any publication that results from it.*

*Before we start, can each of you confirm for the recorder that you agree to the recording of this focus group discussion?*

**[Note to facilitator: Wait for oral confirmation from each participant to begin].**

**Script:** *Thank you again for your willingness to be in this study. The main goal of this discussion today is to better understand your views on long-acting reversible contraception (LARC) and long-acting injectable PrEP (LAI-PrEP). Your opinions will help us develop strategies to introduce these methods in your community effectively.*

*In a group discussion like this, what really helps is when everyone shares openly, not just with me but with each other. There are no right or wrong answers – we want to learn from your different opinions and ideas. Feel free to agree, disagree, or build on what others say. The more back-and-forth conversations we have, the better we’ll understand what works, what doesn’t, and how things can improve. Think of it as a friendly chat where all your input matters.*

*Before we go into the discussion, let us outline some ground rules to make sure we can hear from everyone and include everyone in the chat. Can you suggest some?*

**[Note to facilitator: Ask the group to suggest some ground rules. After they brainstorm some, make sure the following are on the list. Put the list on a flipchart/somewhere visible in the room/FGD space.]**

- *We shall not use names in this discussion. Each of you will be given a number with which you will refer to yourself. For example, you can refer to yourself as three or five.*
- *There are no right or wrong answers to any of our questions.*
- *Share your thoughts and opinions freely.*
- *Maintain the confidentiality of all group members’ responses – do not share anything said during this group discussion outside of the discussion room.*
- *Respect the opinions of your fellow participants. Let us make this a safe and respectful space for people to share their thoughts.*
- *Please put your phones on silent to prevent them from interrupting the recording.*
- *There will be a refreshment break during the discussion, but feel free to signal to me or the note taker should you need to leave the room (e.g., visit the bathroom) during the discussion.*
- *Be open and honest and have fun!*

*If, during our discussion, there are issues or concerns that you would like to talk about, feel free to bring them up. I will take note of them and answer them directly after the discussion. If I cannot answer them, I can refer you to someone who may be able to help.*

***Script:*** *Thanks, let’s get started. Long-acting reversible contraceptives (LARC) are effective birth control options that can last for several years and can be removed whenever needed. Long-acting injectable PrEP (LAI-PrEP) is an antiretroviral medication administered every two to six months and is highly effective in preventing HIV infection. I would first like to hear your thoughts about LARC and LAI-PrEP.*

|  | **Guiding Questions** | **Probes** |
| --- | --- | --- |
| **I** | **Perceptions and Attitudes Toward LARC and LAI-PrEP (Capability, Opportunity, and Motivation)** | |
| 1. | **What do you know about LARC and LAI-PrEP?** | - How did you first hear about these methods? - What do you know about their purpose? - What sources of information influenced your knowledge? - What benefits do you see? - Do you think these methods meet the needs of AGYW in your community? |
| 2. | **How do you feel about using LARC or LAI-PrEP yourself?** | - What motivates or interests you about these methods? |
| 3. | **What influences your willingness to use LARC or LAI-PrEP?** | - Personal health goals? - Family, partner, or peer opinions? - Myths and misconceptions |
| 4. | **What motivates women in your community to use these methods?** | - Convenience and effectiveness. - Dual protection against pregnancy and HIV. - Other motivators? |
| 5. | **What challenges do women face in using these methods?** | - Stigma or judgment from others. - Difficulty accessing services. - Concerns about side effects or safety. - Partner or family opposition. - Other challenges? |
| 6. | **How do community norms influence the adoption of LARC and LAI-PrEP?** | - Cultural beliefs about contraception and HIV prevention. - Role of male partners or elders in decision-making. - Community acceptance of these methods. |
| 7. | **5. How do you think these methods could benefit your community?** | - Reducing unintended pregnancies. - Reducing new HIV infections. - Empowering women to make health decisions. |

***Script:*** *Thanks for this great discussion so far. I would like to hear your ideas on what factors make it difficult or easy for women to access LARC and LAI-PrEP.*

| **II** | **Identifying Barriers and Facilitators** | |
| --- | --- | --- |
| 8. | **What factors make it difficult for AGYW to access and use LARC and LAI-PrEP?** | - Distance to clinics. - Cost. - Availability of trained providers. - Confidentiality concerns. - Support from healthcare providers? - Community education and awareness campaigns? - Partner or peer support? |
| 9. | **What would make it easier for AGYW to access and use these methods?** | - Community outreach programs. - Counseling or peer support groups including peer navigators. - Affordable or free services. |
| 10. | **What challenges might AGYW face when trying to use LARC or LAI-PrEP?** | - Access to clinics or providers? - Concerns about side effects or safety? - Cultural or societal perceptions? |
| 11. | **What role do healthcare providers play in influencing the adoption of these methods?** | - Quality of counseling. - Provider attitudes and biases. - Trust in healthcare workers. |
| 12. | **How can communities be better involved in promoting these methods?** | - Awareness campaigns. - Involvement of community leaders. - Partnerships with schools or religious institutions. |
| 13. | **How do AGYW perceive HIV prevention and contraceptives in general?** | - What are the most trusted methods? - Are there misconceptions about specific methods in general? |
| ***Script:*** *Thanks for this great discussion so far. I would like to hear about your thoughts on how LARC and LAI-PrEP* *compare with other contraceptives and HIV prevention methods.* | | |
| **III** | **Comparisons With Other Methods** | |
| 14. | **How do LARC and LAI-PrEP compare to methods you’ve used or heard about?** | - Condoms, oral contraceptives, or injectables? - PrEP pills or vaginal rings? |
| 15. | **What do you think makes LARC or LAI-PrEP better or worse than other options?** | - Convenience or ease of use? - Level of protection offered? |
| 16. | **How likely are AGYW in your community to recommend LARC or LAI-PrEP to others?** | - What would motivate them to recommend these methods? - Does more information increase recommendations? - What barriers might prevent them? |
| ***Script:*** *Thanks for this great discussion so far. I would like to hear about your ideas for what strategies would help women adhere to LARC and LAI-PrEP.* | | |
| **III** | **Designing Behavior Change Strategies** | |
| 17. | **How prepared do you feel to take steps to use LARC or LAI-PrEP?** | - Seeking information from health providers? - Complying with usage guidelines? |
| 18. | **What strategies would help AGYW adhere to these methods?** | - Reminders (e.g., SMS, apps). - Support from partners or peers. - Regular follow-ups by healthcare workers. |
| 19. | **How can we address stigma around LARC and LAI-PrEP?** | - Educating the community about their benefits - Engaging men and community leaders. - Sharing success stories from users. |
| 20. | **How can communities and healthcare facilities improve service delivery for these methods?** | - Reducing stigma? - Improving access to services? - Reducing waiting times. - Training more providers. - Offering youth-friendly services. |
| ***Script:*** *Before we wrap up, let’s reflect on anything we might have missed.* | | |
| **IV** | **Closing** |  |
| 21. | Is there anything else you would like to share about LARC and LAI-PrEP? | - Any additional thoughts or suggestions? - Anything else we should talk about that we have not yet discussed? - Do you have suggestions for improving community programs? |
| 22. | Do you have any questions for us? | - Clarifications or concerns about the discussion? |

**Script:** *Thank you all for your time and valuable input. Your thoughts will help shape how we introduce LARC and LAI-PrEP in your community Please remember that you agreed to keep experiences and opinions shared in this discussion confidential and to yourself. You may tell others that you were asked to participate in a discussion about LARC and LAI-PrEP, but please don’t share information about the experiences and opinions of the people who have participated here today. Before you leave, my colleague will collect some anonymous information about your background. Thank you again. This concludes our discussion.*

***[Note to facilitator: End the tape recording here]***
