# Supplementary material for: Community Perspectives and Behavior Change Strategies for Integrated Long-Acting Reversible Contraception and Long-Acting Injectable Preexposure Prophylaxis Roll Out for Adolescent Girls and Young Women in Uganda (COMPASS): Protocol for a Mixed Methods Implementation Science Study
Source: JMIR Res Protoc. 2026 Sep 11;15:e88635. doi: 10.2196/88635 (PMC13615377; doi:10.2196/88635)
Supplement: Supplementary file 3 [file resprot_v15i1e88635_app3.doc]

**In-depth Interview (IDI) Guide**

**Study: COMPASS: Community Perspectives and Behavioral Change Strategies on LARC and LAI-PrEP Rollout Among AGYW in Uganda**

IDI Guide for Key Informants

**Objective of IDI**: These interviews will be conducted with different study community stakeholders to understand systemic and community-level barriers, facilitators, cultural norms, and actionable strategies to support the integrated rollout of LARC and LAI-PrEP.

**INSTRUCTIONS for the Interviewers: How to use the IDI Guide**

1. Section headings are noted with blue highlighting
2. Notes to the facilitators are in **[brackets]*.***
3. Text marked as ***Script*** is meant to be read aloud to participants. This text either explains a topic/section to the respondents or provides some information or instruction. The facilitator can then rephrase in their own words or clarify to the participant as necessary.
4. The IDI guide is set up as a table. The first column notes are the guiding questions. Probes are indicated with bullets in the second column. If you find that the participant does not provide much information in response to the primary question, use these probing topics to encourage further discussion. Use the probes to make sure that all issues are addressed but move on when you feel you are starting to hear repetitive information. Note that a participant may provide responses to some questions that may not require the use of some of the probes.
5. The IDI guide is not meant to be used to take notes; please use a separate notes form.
6. To the extent possible, try to maintain a smooth conversational flow. It is up to the interviewer’s discretion if a question should be skipped if that topic has already been covered by responses to questions earlier in the interview. Please ensure that by the end of the discussion, all the key topics/questions have been covered.

**[Note to interviewer: When ready, start the recorder and mention participant PTID, interview type, and date of IDI prior to reading the introductory script.]**

*Before we start, please can you confirm for the recorder that you have already provided written informed consent to take part in this discussion?*

***[Note to interviewer: Wait for oral confirmation to begin.]***

***Script:*** *Thank you for taking the time to speak with me today. I appreciate your willingness to share your experiences as a study participant and your thoughts on the products used in this study. There are no right or wrong answers to any of my questions; I’m here to learn from your opinions and experiences. (I want to know your experiences and opinions about the topics we discuss today). Everything you say will be kept confidential and anonymous (your name will not be directly linked to this interview or any publication that results from it) If you prefer not to answer a question, that’s completely okay—just let me know, and we can move on to the next topic.*

*However, if you do answer, please answer as openly and honestly as you feel comfortable because we really do want to learn from you. To keep your responses confidential, we shall not use names in this discussion. Each of you will be given a number with which you will refer to yourself. For example, you can refer to yourself as number three or number five .*

*If, during our discussion, there are issues or concerns that you would like to talk about, feel free to bring them up; I will note them down and try to answer them directly after the interview or get someone else in our study team to address the questions with you.*

***Script:*** *Great, thanks. Let’s get started. Long-acting reversible contraceptives (LARC) are effective birth control options that can last for several years and can be removed whenever needed. Long-acting injectable PrEP (LAI-PrEP) is an antiretroviral medication administered every two to six months and is highly effective in preventing HIV infection. I would like to hear first about your thoughts on how you or others in your community perceive these options. Have you had any personal experiences or discussions with peers about LARC, oral PrEP, or the newer injectable PrEP?*

|  | **Guiding Questions** | **Probes** | |
| --- | --- | --- | --- |
| ***I.*** | ***Motivation for Service Uptake and Participation*** | | |
| 1. | **What motivates individuals in the community to use LARC and LAI-PrEP services?** | Can you tell us more about the motivation in relation to:   - Personal health concerns (e.g., HIV prevention, family planning) - Influence from partners or peers to use these methods - Role of community leaders and family in encouraging use - Availability of services (e.g., cost, accessibility, convenience) - Support services (e.g., counseling, health checks) - Social factors (e.g., peer pressure, social stigma) - How do AGYW perceive the long-term benefits of LARC and LAI-PrEP for their future health and well-being? | |
| ***II*** | ***Perceived Barriers to the Adoption of Integrated Services*** | | |
| 2. | **What are the major barriers to the adoption of LARC and LAI-PrEP among AGYW in the community?** | Can you explain how these barriers affect the uptake of services?   - Cultural or religious norms around contraceptive use - Misconceptions about the safety or side effects of LARC and LAI-PrEP - Limited knowledge or misinformation about the benefits of these services - Lack of access to trained providers or health facilities - Stigma associated with HIV prevention methods - Financial barriers or logistical challenges to accessing services - Do AGYW feel confident in discussing LARC and LAI-PrEP with partners, family, or healthcare providers? If not, what makes it difficult? - Availability of and access to LARC and LAI-PrEP | |
| 3. | **How do cultural or social norms influence decision-making around contraceptive and HIV prevention methods?** | Can you provide examples of how these norms shape attitudes and behaviors?   - Gender roles and power dynamics within families - The role of the community in shaping health-related decisions - Are there specific community narratives that discourage AGYW from using LARC or LAI-PrEP? | |
| **III** | ***Facilitators for the Uptake of Integrated LARC and LAI-PrEP Services*** | | |
| 4. | **What do you think would make AGYW more likely to use LARC and LAI-PrEP?** | What factors could encourage or motivate uptake?   - Increased awareness and education about LARC and LAI-PrEP benefits - Community engagement and support from leaders - Accessible health services with trained providers - Provision of services at local clinics or community health centers - Financial support or subsidies to reduce costs - Confidentiality and privacy in accessing services | |
| 5. | **What role can peer mentors or youth champions play in supporting AGYW in their decision-making process?** | How can peer mentors help AGYW understand LARC and LAI-PrEP?   - Providing relatable experiences and peer-led education - Offering emotional support and addressing fears or concerns - Connecting AGYW to healthcare services and resources | |
| 6. | **How can healthcare professionals or service providers better support the use of these methods?** | Can you share ideas on what kind of support would be most helpful for AGYW?   - Counseling and education on the benefits and risks of LARC and LAI-PrEP - Regular follow-up and check-ins with service users - Are there gaps in provider training that impact AGYW’s willingness to access services? | |
| **IV** | ***Cultural Norms and Practices Around Reproductive Health and HIV Prevention*** | | |
| 7. | **What cultural or social practices influence the acceptance of LARC and LAI-PrEP in the community?** | How do these practices support or hinder the adoption of these services?   - Gender expectations and the role of women in making health decisions - Attitudes toward sexual activity and prevention in AGYW - Family or community approval of contraceptive use - How do traditional beliefs about fertility and contraception affect AGYW’s decision-making? | |
| 8. | **Are there any community-specific myths or misconceptions about these methods?** | How do these myths affect the willingness of AGYW to use these methods?   - What steps can be taken to address misinformation in the community? - Who are the most trusted sources of information about LARC and LAI-PrEP in the community? | |
| **V** | **Strategies for Implementing Integrated Services** | | |
| 9. | **What strategies can be used to increase the uptake and successful implementation of LARC and LAI-PrEP in your community?** | Can you suggest practical strategies for improving service delivery?   - Community mobilization efforts (e.g., awareness campaigns, community meetings) - Training community health workers and healthcare providers - Collaborative efforts between local authorities, healthcare professionals, and community leaders - Use of digital platforms or peer navigators to promote services - What role can schools and youth-friendly centers play in promoting these services? | |
| 10. | **What role should community leaders play in promoting the use of these services?** | How can leaders contribute to changing perceptions and improving uptake?   - Leading by example and supporting access to services - Providing a platform for open discussions about LARC and LAI-PrEP | |
| 11. | **How can health systems and policies be improved to ensure the sustainability of these services?** | What changes are needed at the policy level to ensure long-term success?   - Policy advocacy for more funding or resources for reproductive health services - Ensuring availability of services in underserved areas - Are there existing policies that hinder | |
| **VI** | **Evaluation and Feedback for Service Improvement** | | |
| 12. | **How can the success of LARC and LAI-PrEP services be measured in the community?** | What indicators would show that the services are meeting the needs of AGYW?   - Increased uptake rates among AGYW - Positive changes in community attitudes toward reproductive health - Improved health outcomes (e.g., reduced rates of unintended pregnancies, HIV incidence) - What personal or community-level changes would indicate that LARC and LAI-PrEP services are becoming more accepted? | |
| 13. | **What feedback mechanisms could be put in place to continually improve service delivery?** | How can we ensure that feedback is collected and acted upon in a timely manner?   - Regular surveys or focus groups to gather opinions from service users - Ongoing dialogue between service providers and community members | |
| **VI** | **Closing for all participants** |  |  |

***Script****: Thank you for taking time to speak with us today and for your participation in this important study, we have reached the end of our interview.*

***[Note to facilitator: End the tape recording here]***
